# MicroRNAs as systemic biomarkers to assess distress in animal models for gastrointestinal diseases

**DOI:** 10.1038/s41598-020-73972-7

**Published:** 2020-10-09

**Authors:** Simone Kumstel, Heike Janssen-Peters, Ahmed Abdelrahman, Guanglin Tang, Ke Xiao, Nicole Ernst, Edgar Heinz Uwe Wendt, Rupert Palme, Nico Seume, Brigitte Vollmar, Thomas Thum, Dietmar Zechner

**Affiliations:** 1 Rudolf-Zenker-Institute of Experimental Surgery, University Medical Center Rostock, Schillingallee 69a, 18057 Rostock, Germany; 2 Institute of Molecular and Translational Therapeutic Strategies (IMTTS), Hannover Medical School, Hannover, Germany; 3 Unit of Physiology, Pathophysiology and Experimental Endocrinology, Department of Biomedical Sciences, University of Veterinary Medicine, Vienna, Austria

**Keywords:** Physiology, Zoology, Biomarkers, Gastroenterology

## Abstract

Severity assessment of animal experiments is mainly conducted by using subjective parameters. A widely applicable biomarker to assess animal distress could contribute to an objective severity assessment in different animal models. Here, the distress of three murine animal models for gastrointestinal diseases was assessed by multiple behavioral and physiological parameters. To identify possible new biomarkers for distress 750 highly conserved microRNAs were measured in the blood plasma of mice before and after the induction of pancreatitis. Deregulated miRNA candidates were identified and further quantified in additional animal models for pancreatic cancer and cholestasis. MiR-375 and miR-203 were upregulated during pancreatitis and down regulated during cholestasis, whereas miR-132 was upregulated in all models. Correlation between miR-132 and plasma corticosterone concentrations resulted in the highest correlation coefficient, when compared to the analysis of miR-375, miR-203 and miR-30b. These results indicate that miR-132 might function as a general biomarker for distress, whereas the other miRNAs were altered in a disease specific manner. In conclusion, plasma miRNA profiling may help to better characterize the level of distress in mouse models for gastrointestinal diseases.

## Introduction

An improvement of animal welfare standards in research is demanded by the general public and provides a sound foundation for scientists to generate valid data^1^. Frist steps to this approach were made by incorporating the 3R-concept^2^, an extensive harm/benefit-analysis^3^ and severity assessment^4^ into the application process of animal experiments. Severity assessment is required in the European Union and presumes a prospective and retrospective grading of procedures and animal models^4^. Essential to correctly perform the latter is evidence-based assessment of distress in laboratory animals. Several parameters, which might be used for distress assessment in rodents, were introduced in the last decade, such as body weight change, clinical scores^5^, voluntary wheel running^6^, nesting behavior^7,8^, burrowing activity^9–11^, concentrations of plasma corticosterone^12,13^ and fecal corticosterone metabolites (FCMs)^14,15^. By applying these parameters a first evidence-based severity grading on a few animal models and experimental procedures was obtained^6,16–18^. However, even these mostly non-invasive and easy to assess measures carry some disadvantages. The clinical scores and assessing nesting behavior might be influenced by observer bias^19^. Strain-specific differences were observed in nesting activity^20^, burrowing behavior^21^, plasma corticosterone^13,22^ and FCMs^23,24^. In addition, corticosterone does not indicate exclusively distress, but is also influenced by other physiological processes such as circadian rhythm^15^, estrus cycle^25^ and sexual arousal^26^. These limitations point out that current distress analysis needs to be optimized, to enable an accurate and objective comparison of different animal models or strains. There is, therefore, a strong need to develop a widely applicable biomarker for animal distress. MicroRNAs (miRNAs) could address this issue. These small ribonucleic acids are about 20 nucleotides and can be detected in various body fluids. As important post-transcriptional regulators they modify gene expression and are involved in physiological and pathophysiological processes. Also used as therapeutic targets, microRNAs are more and more utilized as biomarkers for clinical relevant diseases such as hypertrophic cardiomyopathy^27^ or cancer^28^. MicroRNAs have been detected in many clinically relevant body fluids including serum and plasma^29^. They are known as highly stable molecules with slow turnover rates^28^ and are described as accurate and sensitive diagnostic tools especially at early stages of disease progression when clinical symptoms are still absent or missed in clinical examinations. Technological advancements made it possible to reliably extract microRNAs from body fluids and evaluate their abundance by TaqMan-based qRT-PCR.

The aim of the present study was to discover, if circulating miRNA candidates might function as biomarkers to assess distress of animals during different gastrointestinal diseases. We, therefore, analyzed the expression of 750 different miRNAs via a high-throughput approach in the plasma of mice before and after induction of pancreatitis and validated promising candidates in two additional gastrointestinal pathologies, such as pancreatic cancer and bile duct ligation (BDL). The expression of these miRNAs was further compared to a multimodal distress assessment of all animal models.

## Results

### Distress assessment during gastrointestinal diseases

**Figure 1:**
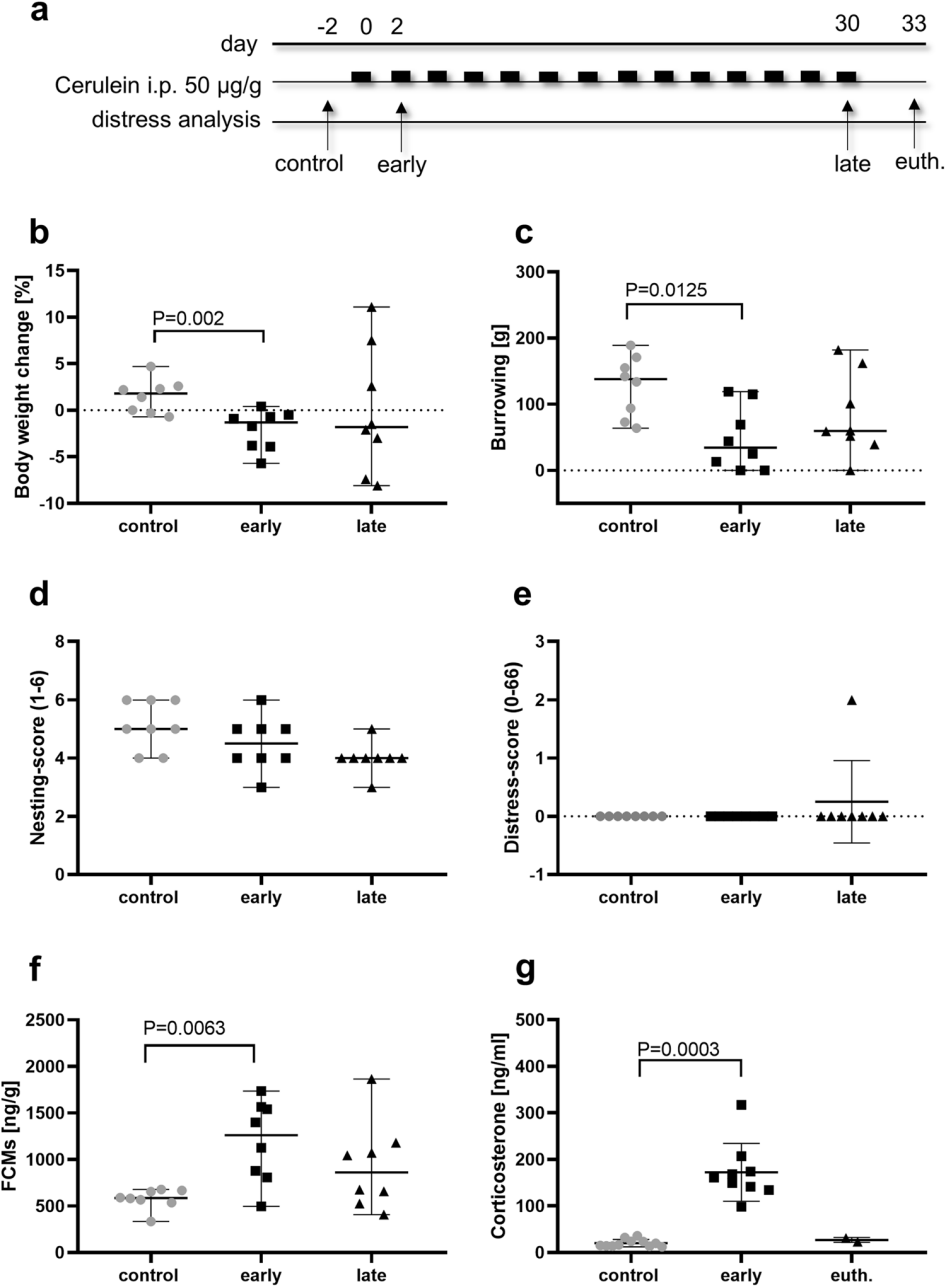
Distress analysis during chronic pancreatitis. Pancreatitis was induced by repetitive, i.p. injections of cerulein, three times a week. The distress was assessed before any intervention (day-2), in the early phase (day 2) and at the late phase (day 30) of disease progression (**a**), by assessing body weight change (**b**), burrowing behavior (**c**), nesting-score (**d**) distress-score (**e**), FCMs (**f**). Just before euthanasia (euth.) corticosterone was assessed in the plasma of mice (**g**). Statistical significance of parametric data was calculated by RM one-way ANOVA, followed by Tukey’s multiple comparisons test (**b**, **c**, **e**). Non-parametric data was analyzed, by Friedman test followed by Dunn’s multiple comparisons test (**d**, **f**) or Kruskal–Wallis test, followed by Dunn’s multiple comparison (**g**). P < 0.05: significant to indicated phase. **a**–**f** (n = 8), g control (n = 11), early (n = 9), late (n = 2).

Pancreatitis was induced by repetitive cerulein injections, three times a week, until euthanasia of the mice. The distress was assessed on these mice before any intervention on day-2, in the early (day 2), and late phase (day 30) of diseases progression, or immediately before euthanasia (euth.) on day 33 (Fig. 1a). The body weight and burrowing activity was significantly reduced during the early phase of pancreatitis (Fig. 1b,c). Non-significant changes were noticed when analyzing nesting activity or the distress-score (Fig. 1d,e). Corticosterone was significantly increased during the early phase of pancreatitis, indicated either by its metabolites in feces or the plasma concentration (Fig. 1f, g). Four out of six parameters indicated a significant increase of distress during the early phase of disease progression.

**Figure 2:**
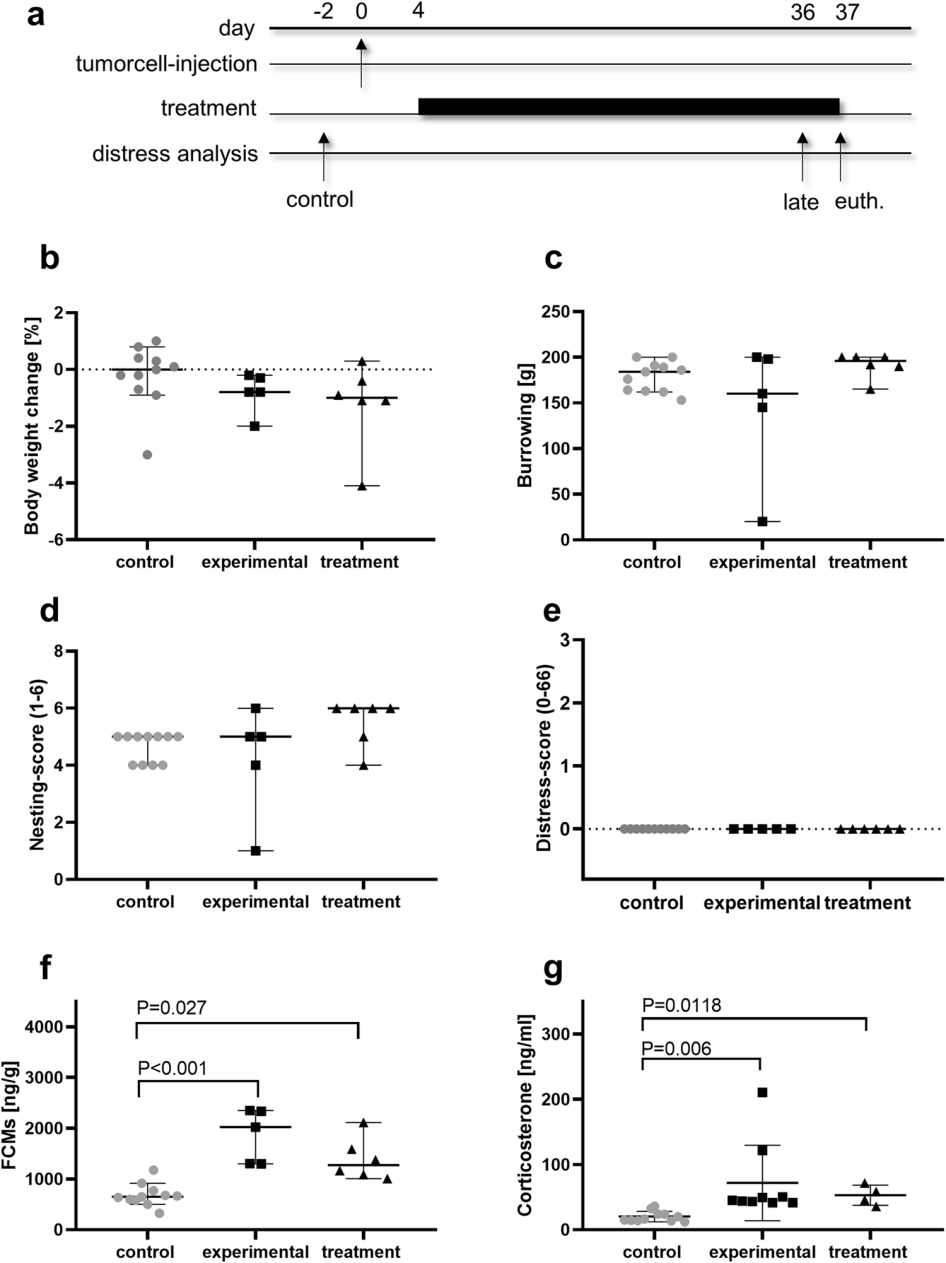
Distress assessment of the murine pancreatic cancer model. Carcinoma cells were injected on day 0 and the animals were treated daily by i.p. injection of metformin and three times a week with galloflavin from day 4 to day 37 (treatment) or the corresponding vehicles, PBS and DMSO (experimental). Distress was assessed before any intervention (day-2) and at the late phase (day 36) of disease progression (**a**), by assessing body weight change (**b**), burrowing behavior (**c**), nesting-score (**d**) distress-score (**e**) and FCMs (**f**) or by assessing plasma corticosterone concentration just before euthanasia (euth.) (**g**). Statistically significant differences of non-parametric data were analyzed by Kruskal–Wallis test, followed by Dunn’s multiple comparisons test (**b**–**e**, **g**). Parametric data was calculated by one-way ANOVA, followed by Tukey’s multiple comparisons test (**f**). P < 0.05 was considered to be significant; **a**–**f**: control (n = 11), experimental (n = 5), treatment (n = 6); **g**: (n = 11), experimental (n = 9), treatment (n = 4).

The syngeneic orthotopic pancreatic cancer model was established by injecting murine carcinoma cells into the pancreas on day 0. The mice were either treated with metformin + galloflavin (treatment), or the corresponding vehicles, PBS + DMSO (experimental), from day 4 until day 37. The distress of mice was analyzed before any intervention on day-2 and during the late phase of disease progression (Fig. 2a). A non-significant reduction of body weight change was observed in the experimental and treatment group at the late phase of disease progression (Fig. 2b). No significant changes were noticed in burrowing, nesting behavior and distress-score (Fig. 2c–e). However, FCMs and plasma corticosterone concentration showed a significant increase at the late phase of cancer progression in the experimental and treatment group (Fig. 2f,g). Only two out of six parameters indicated increased distress at the late phase of disease progression suggesting a need to find additional markers of distress.

**Figure 3:**
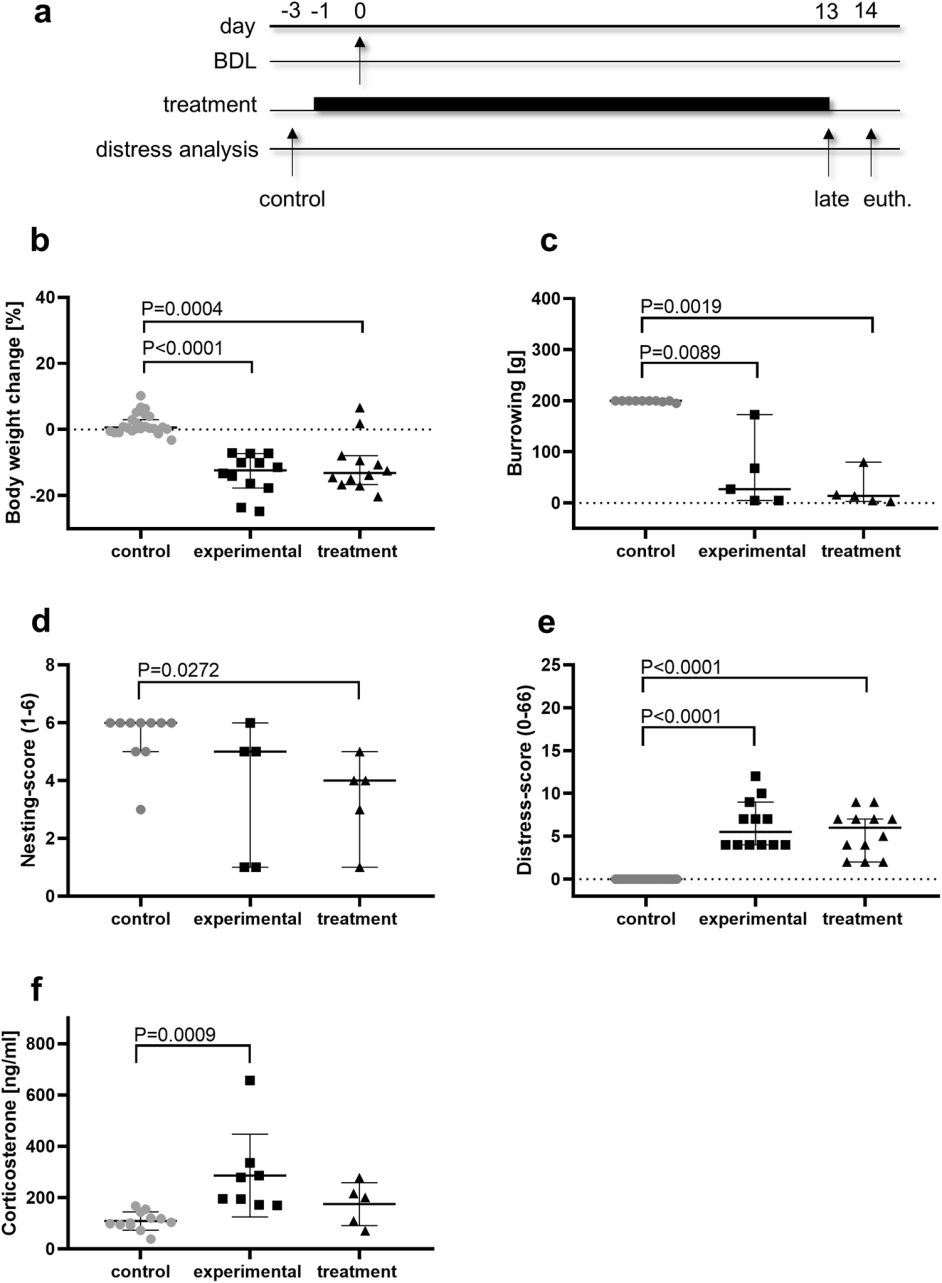
Distress assessment after bile duct ligation. The bile duct ligation was performed on day 0. MCC950 (treatment) or vehicle solution (experimental) were applied as daily i.p. injection from day-1 to day 13. Distress was assessed before any intervention (day-3) and at the late phase (day 13) of disease progression (**a**), by assessing body weight change (**b**), burrowing behavior (**c**), nesting-score (**d**) and a distress-score (**e**) or by assessing just before euthanasia (euth.) plasma corticosterone concentration (**f**). Significant differences of non-parametric data were calculated by Kruskal–Wallis test and Dunn’s method for multiple comparisons (**b**–**f**). P < 0.05 was considered to be significant; **b** + **e**: control (n = 24), experimental (n = 12), treatment (n = 12), **c** + **d**: control (n = 10), experimental (n = 5), treatment (n = 5), **f**: control (n = 12), experimental (n = 8), treatment (n = 5).

BDL was executed on day 0 to induce a cholestatic liver fibrosis. Mice were either injected (i.p.) with the NLRP3 inflammasome inhibitor MCC950 (treatment) or the corresponding vehicle (experimental) on a daily basis from day-1 until day 13 before/after BDL. Distress was evaluated on healthy mice at day-3 and at the late phase of disease progression (Fig. 3a). The body weight change was significantly reduced in the experimental and treatment group with a median body weight loss of − 12 to − 13% (Fig. 3b). The burrowing behavior was also significantly reduced in all mice at the late phase of disease progression (Fig. 3c). We noticed a significant reduction of nesting activity merely in the treatment group (Fig. 3d). At the late phase of BDL score sheet criteria including body weight loss of up to 10%, ruffled fur and dehydration were scored frequently, passive behavior was noticed rarely and ascites was observed once. These observations resulted in a significant increase of distress-score in the experimental as well as the treatment group (Fig. 3e). Plasma corticosterone concentrations were significantly increased in the experimental group (Fig. 3f). All five analyzed distress parameters were significantly altered after BDL.

**Figure 4:**
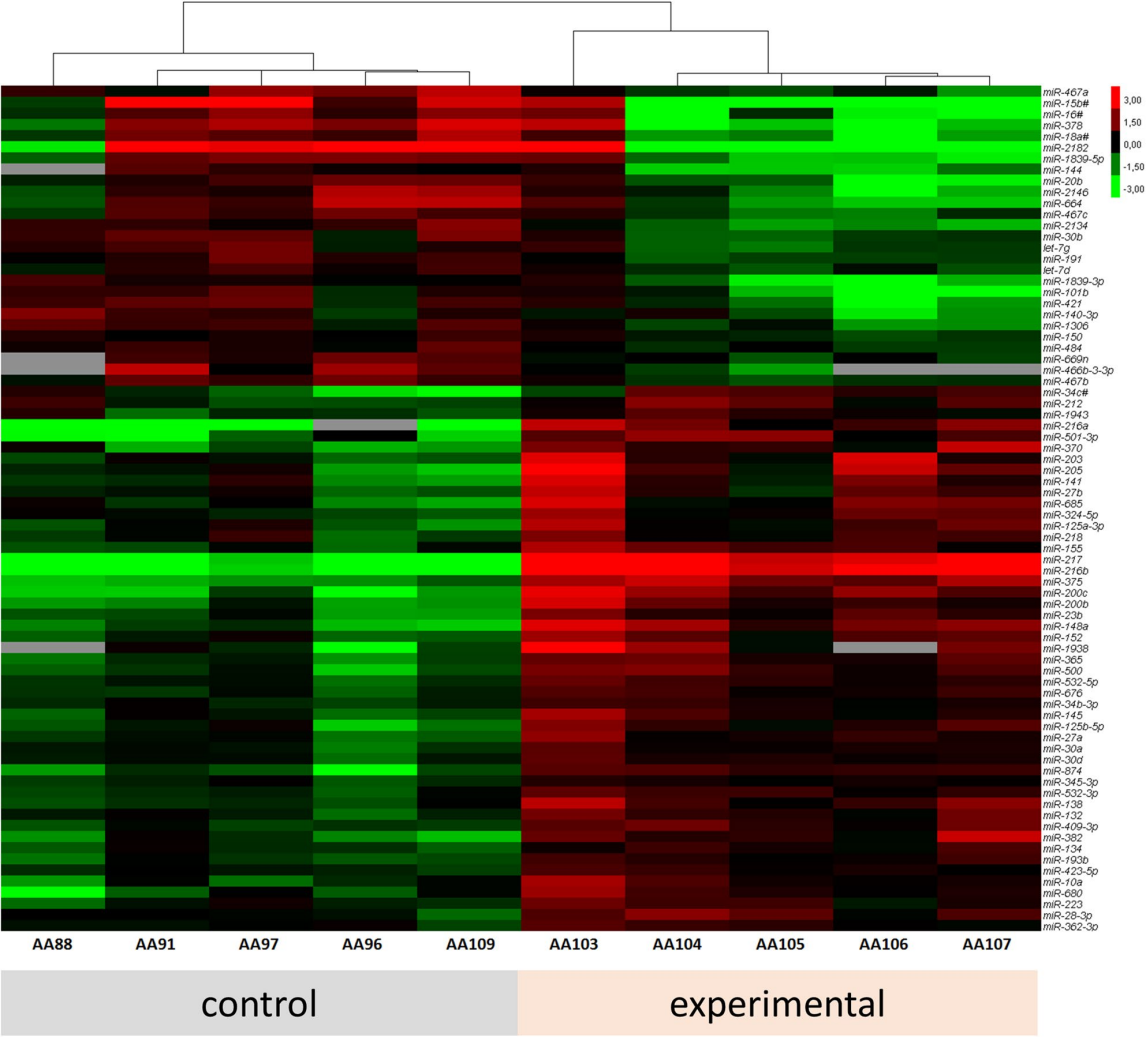
The expression pattern of regulated miRNAs. The heat map illustrates the differentially expressed miRNAs (inclusion criteria: p < 0.05 and mean ct-value per group ≤ 30) in the blood plasma of either healthy mice (control) or mice after induction of pancreatitis at day 2 (experimental); control (n = 5), experimental (n = 5).

**Figure 5:**
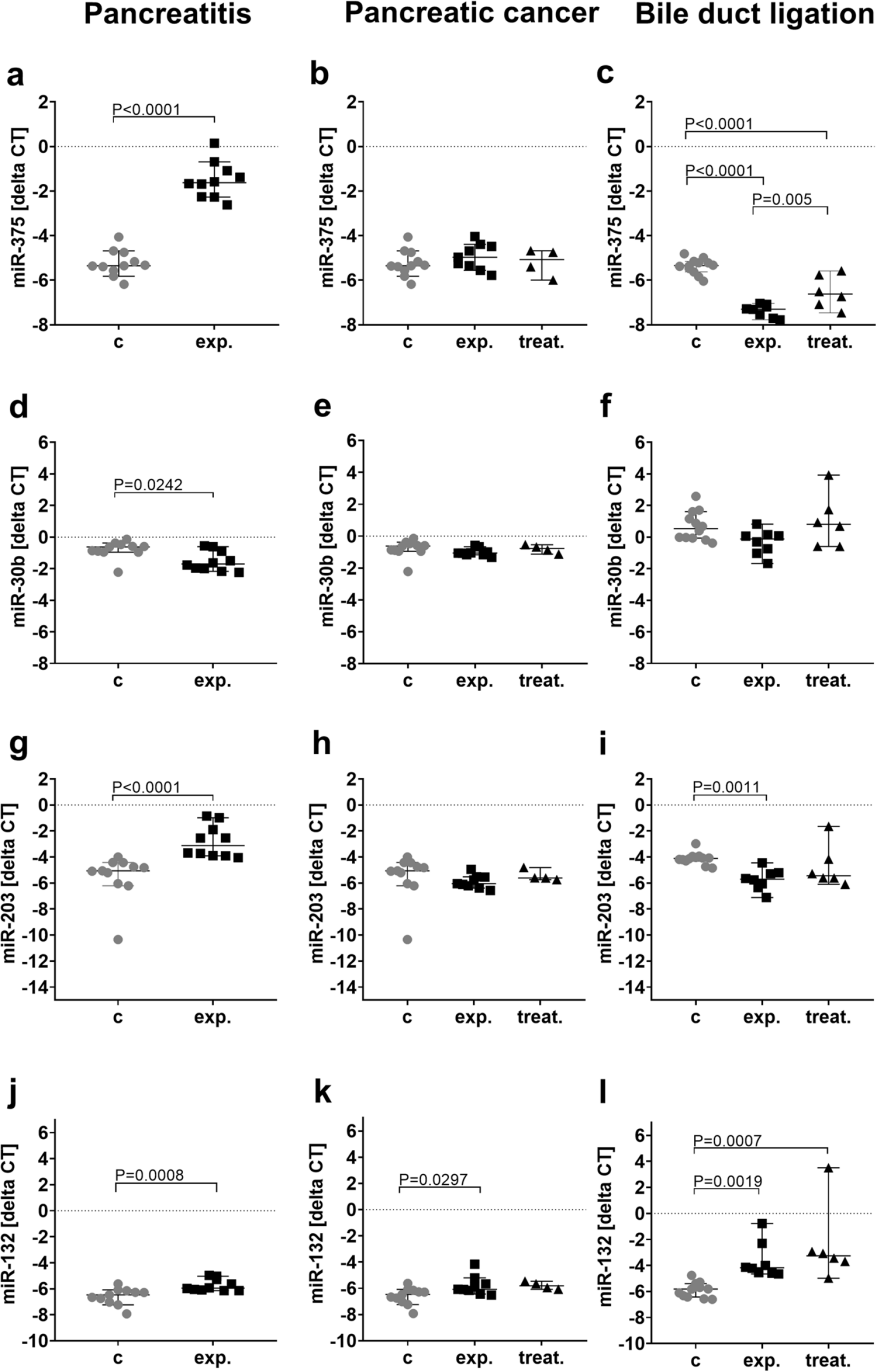
Relative expression of miRNA candidates before and after induction of distinct gastrointestinal diseases in mice. The expression level of miR-375 (**a–c**), miR-30b (**d–f**), miR-203 (**g–i**) and miR-132 (**j–l**) was analyzed in the blood plasma of healthy mice before any intervention (**c**), compared to the early phase of pancreatitis, or at the late phase of pancreatic cancer progression or bile duct ligation, either on mice treated with vehicle (exp.) or undergoing therapy (treat.). Statistically significant differences of non-parametric data were analyzed by Kruskal–Wallis test and Dunn’s method (**e**, **h**, **i**, **l**). Parametric data was calculated by one-way ANOVA, followed by Tukey’s multiple comparisons test (**b**, **c**, **f**, **k**). Parametric data for single comparison was calculated using unpaired t-test (**a**) and non-parametric data was analyzed by Mann–Whitney test (**d**, **g**, **j**). P < 0.05 was considered to be significant. Pancreatitis: **c** (n = 11), exp. (n = 10), Pancreatic cancer: **c** (n = 11), exp. (n = 9), treat. (n = 4); Bile duct ligation: **c** (n = 12), exp. (n = 8), treat (n = 6).

To quantify possible miRNA candidates as biomarker for animal distress, the concentration of 750 miRNAs were analyzed in the blood plasma of mice without any intervention (control) and in the early phase of pancreatitis (experimental). The early phase of pancreatitis was chosen, since the highest distress was measured during that time period (Fig. 1). From 750 miRNAs 76 miRNAs were selected based on inclusion criteria: p < 0.05 and mean ct-value per group below 30 (Fig. 4, Supplementary Figure S1). Out of the 76 miRNAs the three best regulated miRNAs (inclusion criteria: ct-value < 19 and absolute fold change > 1.4, Supplementary Table S1-2) and one miRNA selected by a systemic literature search (Supplementary Figure S2) were selected for further validation. The expression of miR-375 was significantly upregulated in the early phase of chronic pancreatitis (Fig. 5a). In contrast, no significantly changed expression was observed after induction of pancreatic cancer (Fig. 5b). At the late phase after BDL a significant downregulation of miR-375 was quantified in the experimental as well as in the treatment group (Fig. 5c). The expression of miR-30b was only significantly downregulated after pancreatitis induction (Fig. 5d). In contrast, no significant deregulation was observed after induction of either pancreatic cancer or BDL (Fig. 5e,f). The expression of miR-203 was significantly upregulated in the plasma of mice bearing pancreatitis, while no change of expression was noticed in the pancreatic cancer model (Fig. 5g,h). After BDL miR-203 proved to be significantly downregulated in the experimental group (Fig. 5i). MiR-132 was significantly upregulated after induction of pancreatitis, pancreatic cancer and liver fibrosis (Fig. 5j,k).

**Figure 6:**
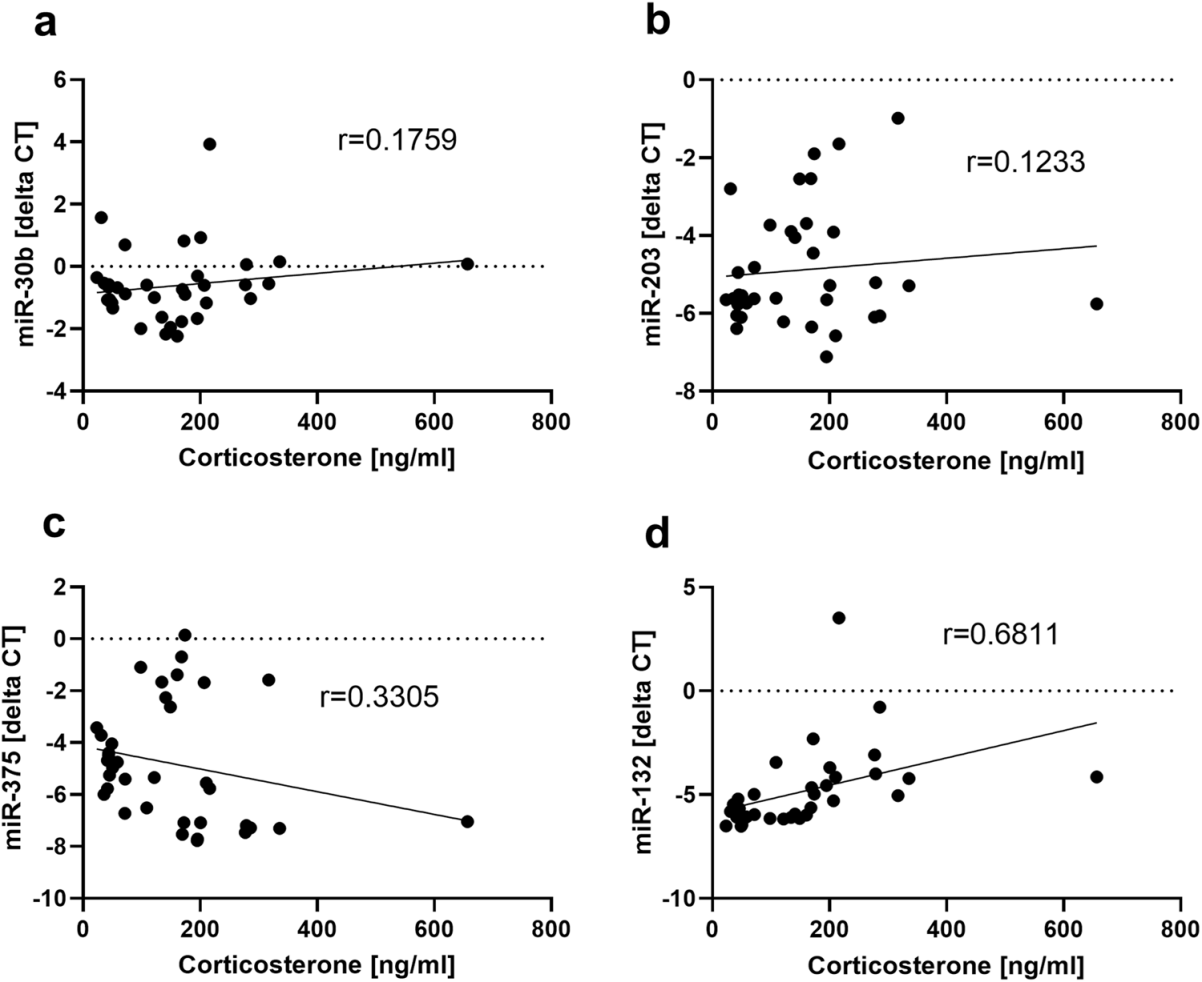
Correlation of plasma corticosterone concentration with miRNAs. Plasma corticosterone concentrations from experimental and treatment cohorts of mice with chronic pancreatitis (early phase), pancreatic cancer and after bile duct ligation (late phase) were pooled and correlated either with the expression of miR-30b (**a**), miR-203 (**b**), miR-375 (**c**) or miR-132 (**d**). Correlation coefficient (r) and p-values were obtained by Spearman correlation. P < 0.05 was considered to be significant (**c**, **d**). **a**–**d** (n = 37).

Additionally, the correlation of plasma corticosterone concentrations with all four miRNA candidates was quantified by pooling data from experimental and treatment mice of all three animal models. Compared to miR-30b (r = 0.1759, p = 0.2977), miR-203 (r = 0.1233, p = 0.4673) and miR-375 (r = 0.3305, p = 0.0457) the highest correlation strength with the individual plasma corticosterone was obtained by miR-132 (r = 0.6811, p < 0.0001; Fig. 6a–d).

**Figure 7:**
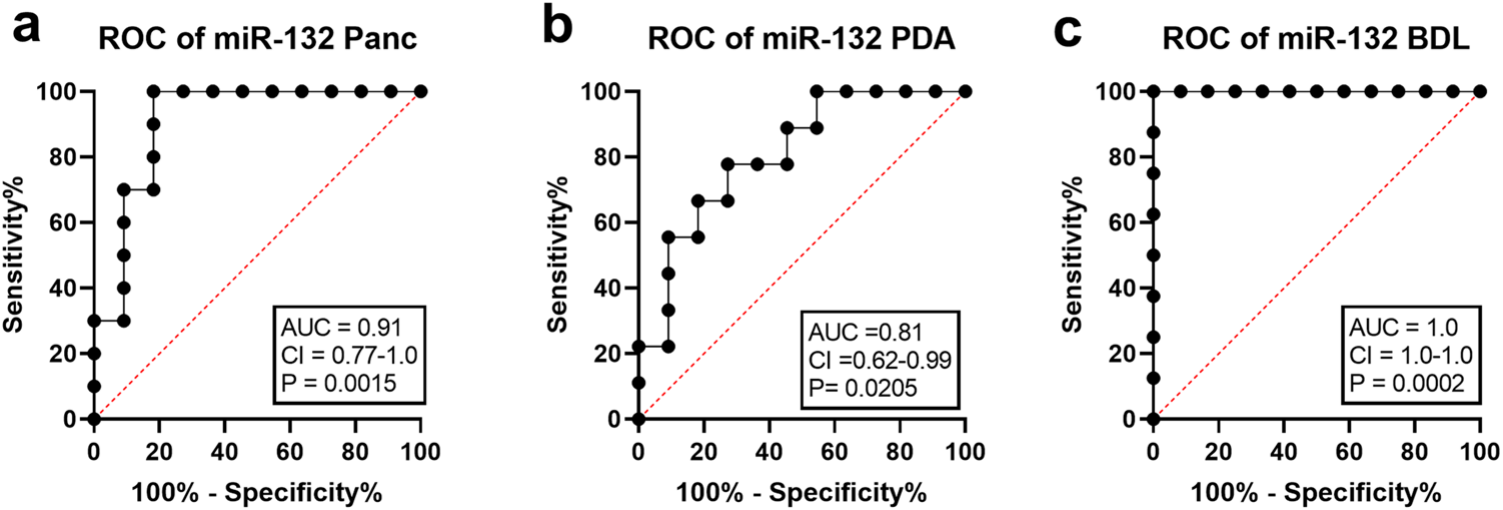
Performance of miR-132 to discriminate between healthy mice and mice after induction of gastrointestinal diseases. The area under the curve (AUC), the 95% confidence interval (CI) and the p-value were assessed by receiver operating characteristic curve analysis on data from healthy mice before any intervention (control) and mice after induction of chronic pancreatitis in the early phase (experimental) (**a**) and the late phase of pancreatic cancer (experimental) (**b**), as well as bile duct ligation (experimental) (**c**). **a**: control (n = 11), experimental (n = 10), **b**: control (n = 11), experimental (n = 9), **c**: control (n = 12), experimental (n = 8).

**Figure 8:**
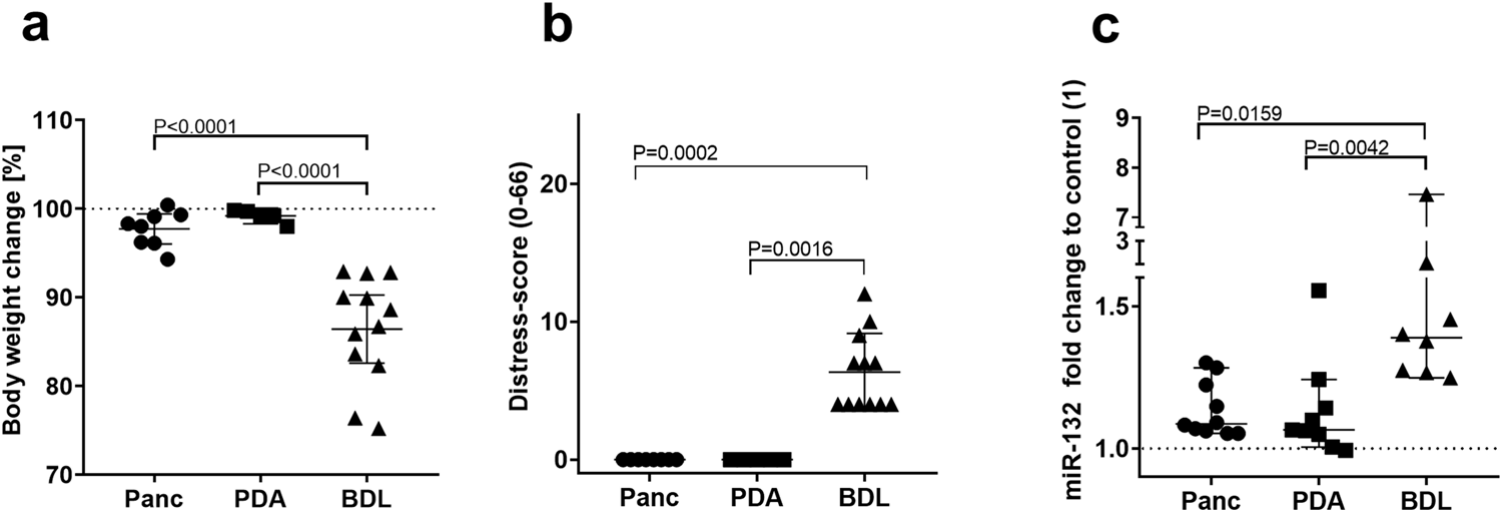
Animal model related distress compared to miR-132 expression. The percentage of body weight change (**a**), and distress-score (**b**), as well as the fold increase of miR-132 expression (**c**) was assessed after induction of pancreatitis (Panc) in the early phase, as well as in the late phase of pancreatic cancer (pancreatic ductal adenocarcinoma, PDA) and bile duct ligation (BDL). Parametric data was calculated by one-way ANOVA, followed by Tukey’s multiple comparisons test (**a**). Statistically significant differences of non-parametric data were analyzed by Kruskal–Wallis test and Dunn’s method (**b**, **c**). P < 0.05 was considered to be significant. **a**, **b**: Panc (n = 8); PDA (n = 5), BDL (n = 12); **c**: Panc (n = 10), PDA (n = 9), BDL (n = 8).

The performance of miR-132 to differentiate between healthy mice (control) and mice after disease induction (experimental) was analyzed by ROC-curve analysis. ROC-curve analysis is used to graph the performance of a diagnostic tests, an AUC of 1.0 represents high discriminatory power, while a value of 0.5 demonstrates no discriminatory power^30^. MiR-132 indicated a high discriminatory power after pancreatitis (AUC = 0.91, CI 0.77–1.0) and pancreatic cancer induction (AUC = 0.81, CI 0.62–0.99; Fig. 7a,b). In the liver fibrosis model miR-132 exhibited an even better performance to discriminate between healthy and bile duct ligated mice with an AUC of 1.0 (CI 1.0–1.0, Fig. 7c). In order to compare all three animal models body weight change, distress-score and miR-132 expression level were evaluated. After BDL the mice lost significantly more body weight compared to the pancreatic diseases (Fig. 8a). The distress-score after BDL was also significantly increased compared to the pancreatitis and the pancreatic cancer model (Fig. 8b). In accordance with the altered distress parameters, a significantly higher upregulation of miR-132 expression was observed after BDL, when compared to the pancreatitis and the pancreatic cancer model (Fig. 8c).

## Discussion

The present study revealed that the expression of many different miRNAs is modulated after induction of various gastrointestinal diseases in mice. MiR-132 proved to be significantly upregulated in all gastrointestinal animal models and showed also a significant correlation with individual plasma corticosterone concentrations. The most intense upregulation of miR-132 was detected after bile duct ligation, while the induction of pancreatitis and pancreatic cancer resulted in a more moderate elevation. This was consistent with higher distress caused by BDL when compared to distress caused by pancreatitis and pancreatic cancer. These results indicate that miR-132 might function as a good biomarker to assess distress in gastrointestinal animal models, whereas miR-375, miR-30b and miR-203 might be biomarkers indicating disease-specific pathophysiological processes. For example, miR-375 was specifically induced during pancreatitis and this miRNA is known to be upregulated in patients and animals bearing chronic pancreatitis and might, therefore, reflect the degree of the pancreatic injury^31–34^. After BDL miR-375 expression was significantly downregulated. This result is consistent with reduced concentration of miR-375 in serum samples of patients suffering from Hepatitis C^35^, nonalcoholic fatty liver disease^36^ or hepatocellular carcinoma^37^. All these pathologies are associated with hepatic fibrosis. The expression of miR-203 was induced during pancreatitis in mice and was also found to be upregulated in patients with acute pancreatitis and in a cerulein treated rat pancreatic acinar cell line^38^. This study also suggested that a high level of miR-203 aggravates the cerulein induced cell injury through suppression of nuclear factor interleukin-3^38^. In line with our results, a significant downregulation of miR-203 was also observed in mice after induction of alcoholic induced liver damage^39^.

Out of the 4 miRNA candidates exclusively miR-132 was significantly upregulated after induction of all gastrointestinal diseases (Fig. 5j–l). The most intense increase of miR-132 was noticed after bile duct ligation, while pancreatitis and pancreatic cancer resulted in a low to moderate upregulation of miR-132. These animal model specific increases of miR-132 were consistent with the elevation of distress confirmed by the multimodal distress assessment (Figs. 1–3, 8). BDL resulted in a significant alteration of all assessed parameters. Especially a high body weight loss (median of 13%) and a significant increase of the distress score (median of 6) indicated high distress of mice at the late phase of this pathology. However, a limitation of this study is that we have used BALB/c mice for BDL and C57Bl/6 J mice for the pancreatic cancer and pancreatitis model. The BALB/c strain was chosen for the BDL model, since it was reported that liver injury causes marked hepatic fibrosis in this mouse strain^40–42^. The BALB/c strain is, however, also known to be more sensitive to some stressors when compared to the C57BL6/J mice^13,22,23^. This difference is probably caused by higher anxiety or genetic alterations of the HPA axis^22,23^. Thus, the observed differences in distress parameters and miR-132 expression might not only be influenced by the severity of distinct interventions, but also by strain specific differences in the sensitivity to insults. Both aspects might contribute to distress and higher miR-132 concentration in the BDL model, when compared to pancreatitis and pancreatic cancer.

The level of distress when comparing pancreatic cancer, pancreatitis and BDL was perfectly reflected by the upregulation of miR-132 (Fig. 8). In all three animal models miR-132 had a very high performance as diagnostic test when differentiating between control animals and animals suffering from pancreatitis, pancreatic cancer or BDL (Fig. 7). These results might indicate that miR-132 might function as a possible biomarker to assess distress of animals. An upregulation of miR-132 was also observed in other studies after different stressors in mice and rats, such as predator scent induced anxiety^43^, chronic restrain stress^44^, neuropathic pain^45^, trace fear conditioning^46^, acute and chronic stress^47^. The above mentioned literature points out that miR-132 expression is able to respond to the multidimensional nature of animal distress, including anxiety, pain, psychological and physical stress. The stress induced upregulation of miR-132 leads to a suppression of hippocampal acetylcholinesterase (AChE) level, associated with an improved cognitive function after stress induction^43,48^. A long-lasting suppression of AChE might even reduce stress induced neuronal sprouting^43,48^. Through the same target miR-132 is also able to restrict inflammation^49^. The upregulation of miR-132 might therefore be a coping mechanism for distress.

Besides an upregulation of miR-132 in response to distress, some studies also observed a significant downregulation of miR-132 in response to social and physical enrichment^50,51^. This result implies that the upregulation of miR-132 might function as biomarker for distress and that its downregulation indicates well-being of animals.

The expression of miR-132 in response to stress was quantified primarily in tissue, such as hippocampus^43,44,46,47,50,52,53^, spinal cord^54^ or dorsal root ganglia^55^. In contrast to the mentioned literature, the present study proved that the stress-specific deregulation of miR-132 is not just locally restricted to neuronal tissue, but can also be quantified systemically in the blood. This is an important point when using miR-132 as biomarker for distress in future studies.

One limitation of the present study is that these results do not indicate the source of circulating miR-132 expression. The increase of miR-132 might be caused by a stress-specific increase in the neuronal tissue^43–45^, however we cannot completely exclude that the increased systemic miR-132 level is caused by damage of the pancreas or liver. No link between miR-132 expression and pancreatitis was reported so far. However, miR-132 expression is reported to be upregulated in beta cells of the pancreas in response to hyperglycemia and obesity^56,57^. In previous studies we could prove that hyperglycemia is not observed during a cerulein induced pancreatitis^58,59^. This suggests that miR-132 expression is not induced by a pancreatogenic diabetes mellitus secondary to chronic pancreatitis. The connection of miR-132 expression and pancreatic cancer is controversially discussed in the literature, some studies reported a downregulation of the miR-132 expression in cancer tissue and cells^60–62^, while other studies confirmed an upregulation of miR-132^63–65^. The hepatic expression of miR-132 is reported to be upregulated after alcoholic hepatitis^66^, nonalcoholic fatty liver disease (NAFLD), hepatic steatosis^67^, as well as cholestasis^68^. Zong et al. found even a correlation between the serum miR-132 expression and the NAFLD risk in patients^69^. According to the above mentioned literature a possible liver-disease-specific regulation on the systemic miR-132 expression cannot be eliminated completely. However, in the present study we did not observe a significant correlation between miR-132 expression and liver fibrosis (% of liver section area stained positively for collagen 1 alpha, r = 0.2133, p = 0.6615) or necrosis (% of necrotic area of H/E stained liver sections, r = 0.03571, p = 0.9635) after bile duct ligation. In addition, the significant increase of miR-132 expression after pancreatitis and pancreatic cancer (Fig. 5j–k) might also argue against the liver damage as a sole cause for miR-132 expression, since the liver function is not affected in these animal models. These results in combination with the significant correlation between miR-132 and individual plasma corticosterone concentration (Fig. 6d), support our hypothesis that miR-132 might function as systemic biomarker for distress in animal models for gastrointestinal diseases.

## Methods

### Ethical statement and animals

All animal experiments were approved by the local authority (Landesamt für Landwirtschaft, Lebensmittelsicherheit und Fischerei; 7221.3-1-002/17; 7221.3-1-019/15). The experiments were performed in accordance with the German law^70^ for animal protection and the European Union Directive 2010/63/EU^4^. Breeding pairs of C57Bl/6 and BALB/c mice were originally purchased from Charles River and further bred in our facility in Rostock under specific pathogen free conditions. During the experiment the mice were housed in European standard type III cages (Zoonlab GmbH, Castrop-Rauxel, Germany) at a 12 h dark/light cycle (dark phase: 7 pm–7 am) and a temperature of 21 ± 2 °C, with a relative humidity of 60 ± 20%. Food pellets (ssniff Spezialdiäten GmbH, Soest, Germany) and tap water was provided ad libitum. Enrichment was provided by nesting material (Zoonlab GmbH), paper role (ssniff Spezialdiäten GmbH) and wooden block (Abedd Vertriebs GmbH, Vienna, Austria).

### Induction of gastrointestinal diseases and treatments

Chronic pancreatitis was induced in 21 male 9–15 weeks old C57Bl/6 mice by three repetitive i.p. injection of cerulein (50 µg/kg; Merck, Darmstadt, Germany), diluted in 0.9% sodium chloride, at a rate one every hour, three times a week up to 30 days, the mice were euthanized on days 33 after the first cerulein injection.

For the orthotopic injection of cancer cells, 18 male 12–14 weeks old C57Bl/6 mice were anaesthetized with 1.2–2.5% isoflurane, placed on a warming plate and the eyes were kept wet by eye ointment. The mice were shaven, a laparotomy was performed and murine 6606PDA cells (2.5 × 10^5^ cells in 5 µl matrigel) were injected into the pancreas, using a 25 µl syringe (Hamilton Syringe, Reno, NV, USA). The abdomen was closed using coated 5–0 vicryl suture (Johnson & Johnson Medical GmbH, New Brunswick, NJ, USA) for the peritoneum and a 5–0 prolene suture (Johnson & Johnson Medical GmbH). The surgery lasted up to 20 min for each mouse. The mice were then allocated in a non-random manner matching the performance of the behavior tests into the distinct treatment groups. The chemotherapeutic intervention was conducted by daily intraperitoneal injection of metformin (125 mg/kg in PBS; Merck, Darmstadt, Germany) in combination with galloflavin (20 mg/kg in DMSO, Tocris Bioscience, Bristol, United Kingdom), which was injected three times a week. The sham treatment was performed using an equivalent volume of the corresponding vehicle (PBS, DMSO).

For BDL 24 male BALB/c mice (age: 10–21 weeks) were anaesthetized with 1.2–2.5% isoflurane, placed on a heating plate and eye ointment was applied. The abdomen was shaven and a midline laparotomy was performed. The bile duct was ligated three times with 5–0 silk and transected between the two distal ligations. The peritoneum and skin was closed with 5–0 prolene suture (Johnson & Johnson Medical GmbH). The surgery lasted 15–25 min for each mice. The treatment was executed by daily intraperitoneal injection of NLRP3 inflammasome inhibitor MCC950 (20 mg/kg in aqua, Sigma Aldrich, St. Louise, USA) or the corresponding vehicle from day-1 until day 13 after BDL.

As perioperative analgesia for BDL and carcinoma cell injection 5 mg/kg carprofen (Rimadyl, Pfizer GmbH, New York City, USA) was injected subcutaneously before each surgery. For all mice 1250 mg/l metamizol (Ratiopharm, Ulm, Germany) was provided as analgesia in the drinking water from the first day of intervention until euthanasia.

### Distress assessment

The body weight was assessed 24 h after the indicated days before any intervention, early or late phase of disease progression, to allow sufficient time for body weight adjustments. The body weight change was calculated as percentage of the value assessed 3–5 days before any intervention. The distress score was assessed on a daily basis for health monitoring, according to a previously published score sheet^12,71^. The burrowing behavior was performed according to Deacon^9,10^, by placing a burrowing tube, filled with 200 ± 1 g pellets into the home cage 2–3 h before the dark phase. The burrowed amount of pellets (ssniff Spezialdiäten GmbH) was calculated for the C57Bl/6 mice after 2 h (6-7 pm) and for the BALB/c strain after 17 h on the next morning. The nesting activity was assessed by providing a nestlet (5 cm square of pressed cotton batting, Zoonlab GmbH), in the home cage 30 to 60 min before the dark phase. The nest was scored on the next morning, additional to the 5-point scale from Deacon we scored 6-points for a perfect nest, which looked like a crater and more than 90% of the circumference was higher than the body height of a mouse. Nesting and burrowing behavior was assessed two times in group housing, since the learning process is enhanced by social facilitation^9^, afterward the animals were single housed until euthanasia. Mice which burrowed less than 10 g during the pre-experimental phase were excluded for further distress analysis (4 C57Bl/6 mice). To analyze FCMs, 200–400 mg feces were collected after 24 h of the indicated days for each animal model from the home cage. Feces were dried for 4 h at 65 °C and stored at − 20 °C. 50 mg of dried feces were extracted with 80% methanol and were analyzed by a 5α-pregnane-3β,11β,21-triol-20-one enzyme immunoassay^15^. FCMs were not analyzed after BDL, because steroid hormones are known to be excreted via the bile duct^72^. Some of the data from the distress assessment was already published in other contexts^16,71,73^.

### Blood and tissue sampling

To analyze the expression of miRNAs, an amount of 100 µl blood plasma was necessary from each mouse. Since a huge blood loss (> 200 µl), would lead to long-lasting impairment of the mice, the blood was taken only once and the animals were euthanized afterwards. Thus, 11 C57Bl/6 mice (10–12 weeks old) and 12 BALB/c mice (8–12 weeks old) were used as control animals (experiencing no intervention before the blood collection). In order to analyze blood plasma during the early phase of pancreatitis, 10 additional C57Bl/6 mice (12–14 weeks old) were bled and sacrificed on day 2. For the blood sampling the mice were anesthetized for 2–3 min by 5% isoflurane and 500–700 µl blood was collected via retro orbital puncture within 30 s, followed by immediate euthanasia via cervical dislocation. For the quantification of plasma, plasma of all mice, used for the miRNA expression analysis was measured in a blinded fashion with the mouse and rat ELISA-Kit (DEV9922, Demeditec Diagnostics GmbH, Erfurt, Germany) according to manufacturer instructions. To quantify the degree of liver damage, four micrometers thick paraffin-embedded liver slices were stained with hematoxylin and eosin (H/E), as well as immunostained with rabbit -anti-collagen 1α (1:200, Abcam 34719, Cambridge, UK) and HRP conjugated goat-anti-rabbit (1:100, Dako Deutschland GmbH, Hamburg, Germany) antibodies.

### Analysis of miRNAs

For assessing miRNAs, the RNA was isolated by miRNeasy plasma Mini Kit (Qiagen) according to instructions for small RNA isolation. 30 μl of RNA-solution were obtained from an amount of 100 μl plasma. Synthetic Caenorhabditis elegans miR-39 (5 μl of 1 fmol/μl) was added as spike-in control during RNA isolation to the Qiazol/chloroform/plasma mixture. For performing miRNA expression profile we selected one out of three animal-models (pancreatitis model) and randomly chose 5 samples per group. After preamplification, TaqMan gene expression array cards (Rodent A and B Card Set v3, Thermo Fisher Scientific, Waltham, Massachusetts, USA) were used according to manufacturer’s instructions using ViiA 7 Real-Time PCR System with QuantStudio Real-Time PCR Software v1.3. 750 different reactions of highly conserved miRNAs were measured. Screening results were normalized using global normalization. For analyzing these array data, we used -ddCT method. From these 750 miRNAs, 76 significantly deregulated miRNAs were presented as heat map in Fig. 4 and in the volcano plot Supplementary Figure S1 (inclusion criteria: p < 0.05 and mean ct-value per group below 30). Out of the 76 significantly deregulated miRNAs, miR-375, miR-30b and miR-203 were selected for further validation The selection process can be comprehended by sorting the 76 miRNAs ascending according to the ct-values (Supplementary Table S1), the 9 miRNA candidates with the lowest ct-value (ct-value < 19) were further sorted according to the absolute log FC (Supplementary Table S2) and the three miRNAs with the highest absolute log FC (> 1.4) were chosen for further quantification. Additionally, we chose miR-132 for further validation due to a systematic literature review (Supplementary Figure S2). This review was performed according to the PRISMA guidelines^74^. Publications were identified by searching PubMed, on 7 May 2020, using the following search strategy: (miR [tiab] OR miRNA [tiab] OR microRNA [tiab]) AND regulation [tiab] AND (animal [tiab] OR rodents [tiab] OR mice [tiab] OR mouse [tiab] OR rat [tiab]) AND (stress [tiab] NOT oxidative NOT hypoxic NOT metabolic NOT mechanic NOT endoplasmic reticulum OR distress [tiab] OR "severity assessment" [tiab] OR pain [tiab] OR suffering [tiab] OR anxiety [tiab] OR harm [tiab]) Exclusion criteria: We excluded article types, which were reviews or commentaries; literature written not in English and irrelevant literature (literature which does not meet the inclusion criteria). Plasma samples out of all three animal models were chosen for validation process. TaqMan MicroRNA Reverse Transcription Kit (Applied Biosystems, Foster City, California, USA) was used according to developer’s instructions to transcribe the isolated RNA of each blood sample to their complementary DNA (cDNA).

### Data analysis

For graphs and statistical analysis, the program GraphPad Prism 8.0 (GraphPad Software, San Diego, USA) was used. For Figs. 1–3, 5 and 8 the data is presented in form of point plots, indicating median ± 95% confidence interval. For the assessment of normality, the Shapiro–Wilk-test was applied and for scores (nest-score, distress-score) the Kolmogorow-Smirnov test. Significant differences were calculated in case of paired samples (Fig. 1) for parametric data by RM one-way ANOVA, followed by Tukey’s multiple comparisons test. Non-parametric data was analyzed, by Friedman test followed by Dunn’s multiple comparisons test. In case of unpaired samples (Figs. 2, 3, 5), parametric data was calculated by one-way ANOVA and Tukey’s multiple comparisons test. Non-parametric data was analyzed by Kruskal–Wallis test and Dunn’s method for multiple comparisons. Parametric data for single comparison was calculated using unpaired t-test and non-parametric data was analyzed by Mann–Whitney test. Differences with P < 0.05 were considered to be significant. Correlation analysis were performed with Spearman’s rank correlation coefficient, since not just a linear but also a monotonic relation between the variables might be possible. To characterize the performance of miRNA to quantify mice before and after disease induction, receiver operating characteristic (ROC) curve analysis was performed and the area under the curve (AUC), p-value and 95% confidence intervals were calculated. The exact sample size for each figure is indicated in the figure legends.

## Supplementary information

Supplementary information.

## Data Availability

The datasets used and/or analyzed during the present study are available from the corresponding author on reasonable request.
