# Supplementary material for: MicroRNAs as systemic biomarkers to assess distress in animal models for gastrointestinal diseases
Source: Sci Rep. 2020 Oct 9;10:16931. doi: 10.1038/s41598-020-73972-7 (PMC7547723; doi:10.1038/s41598-020-73972-7)
Supplement: Supplementary file 1 — Supplementary information. [file 41598_2020_73972_MOESM1_ESM.pptx]

## Slide 1
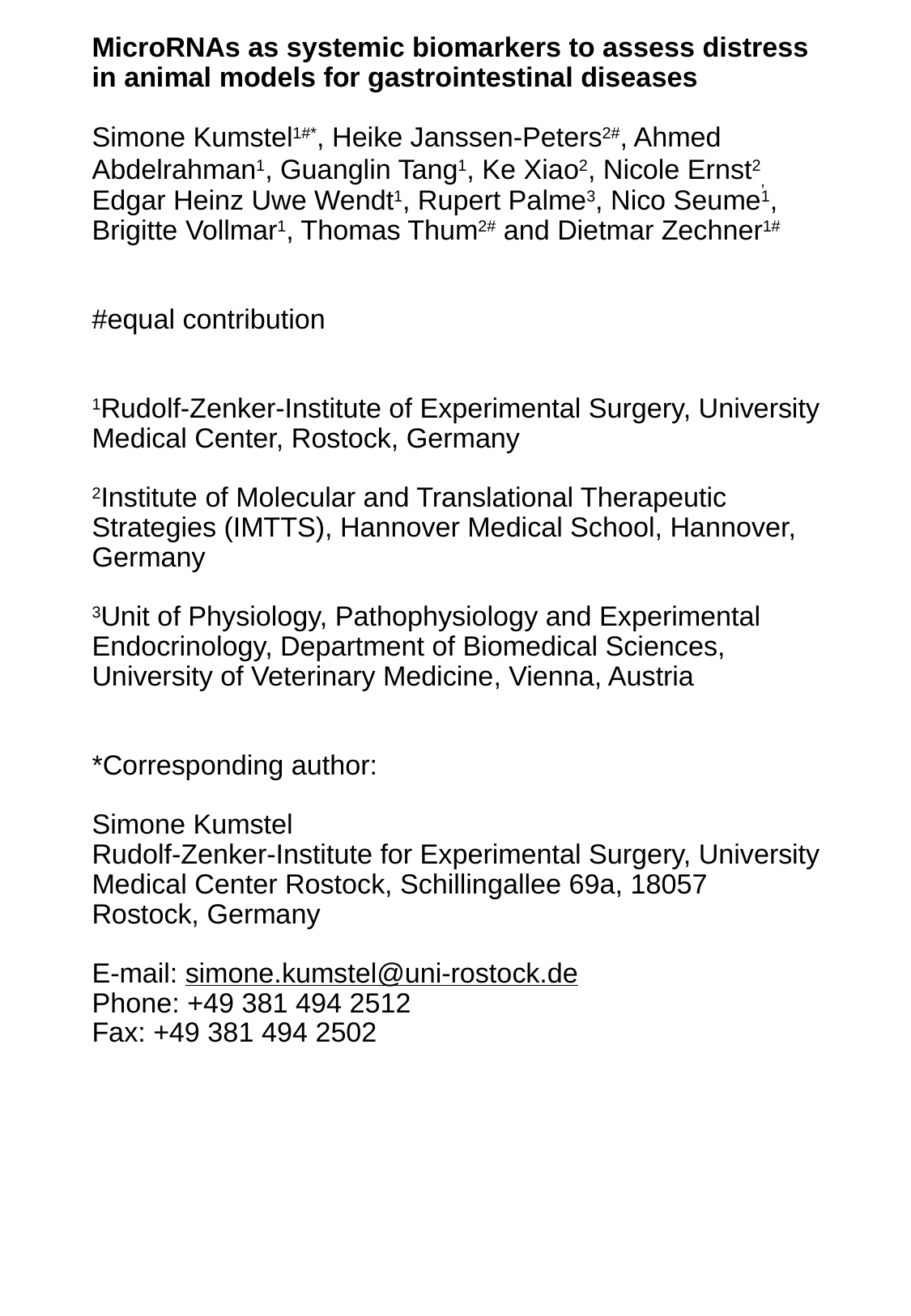

# MicroRNAs as systemic biomarkers to assess distress in animal models for gastrointestinal diseases   Simone Kumstel1#*, Heike Janssen-Peters2#, Ahmed Abdelrahman1, Guanglin Tang1, Ke Xiao2, Nicole Ernst2, Edgar Heinz Uwe Wendt1, Rupert Palme3, Nico Seume1, Brigitte Vollmar1, Thomas Thum2# and Dietmar Zechner1#   #equal contribution 1Rudolf-Zenker-Institute of Experimental Surgery, University Medical Center, Rostock, Germany   2Institute of Molecular and Translational Therapeutic Strategies (IMTTS), Hannover Medical School, Hannover, Germany   3Unit of Physiology, Pathophysiology and Experimental Endocrinology, Department of Biomedical Sciences, University of Veterinary Medicine, Vienna, Austria   *Corresponding author: Simone Kumstel Rudolf-Zenker-Institute for Experimental Surgery, University Medical Center Rostock, Schillingallee 69a, 18057 Rostock, Germany E-mail: simone.kumstel@uni-rostock.de Phone: +49 381 494 2512 Fax: +49 381 494 2502

## Slide 2
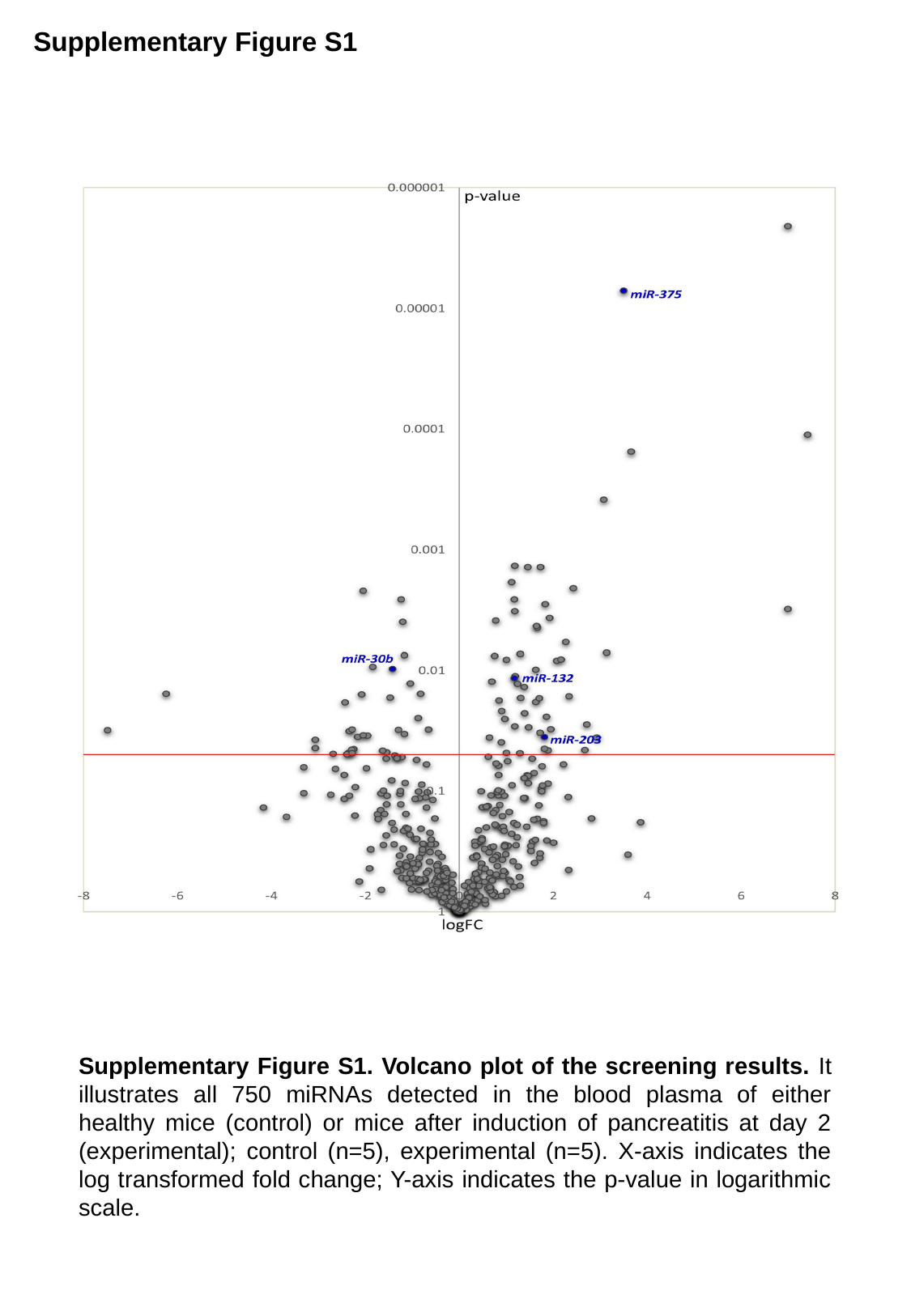

Supplementary Figure S1
Supplementary Figure S1. Volcano plot of the screening results. It illustrates all 750 miRNAs detected in the blood plasma of either healthy mice (control) or mice after induction of pancreatitis at day 2 (experimental); control (n=5), experimental (n=5). X-axis indicates the log transformed fold change; Y-axis indicates the p-value in logarithmic scale.

## Slide 3
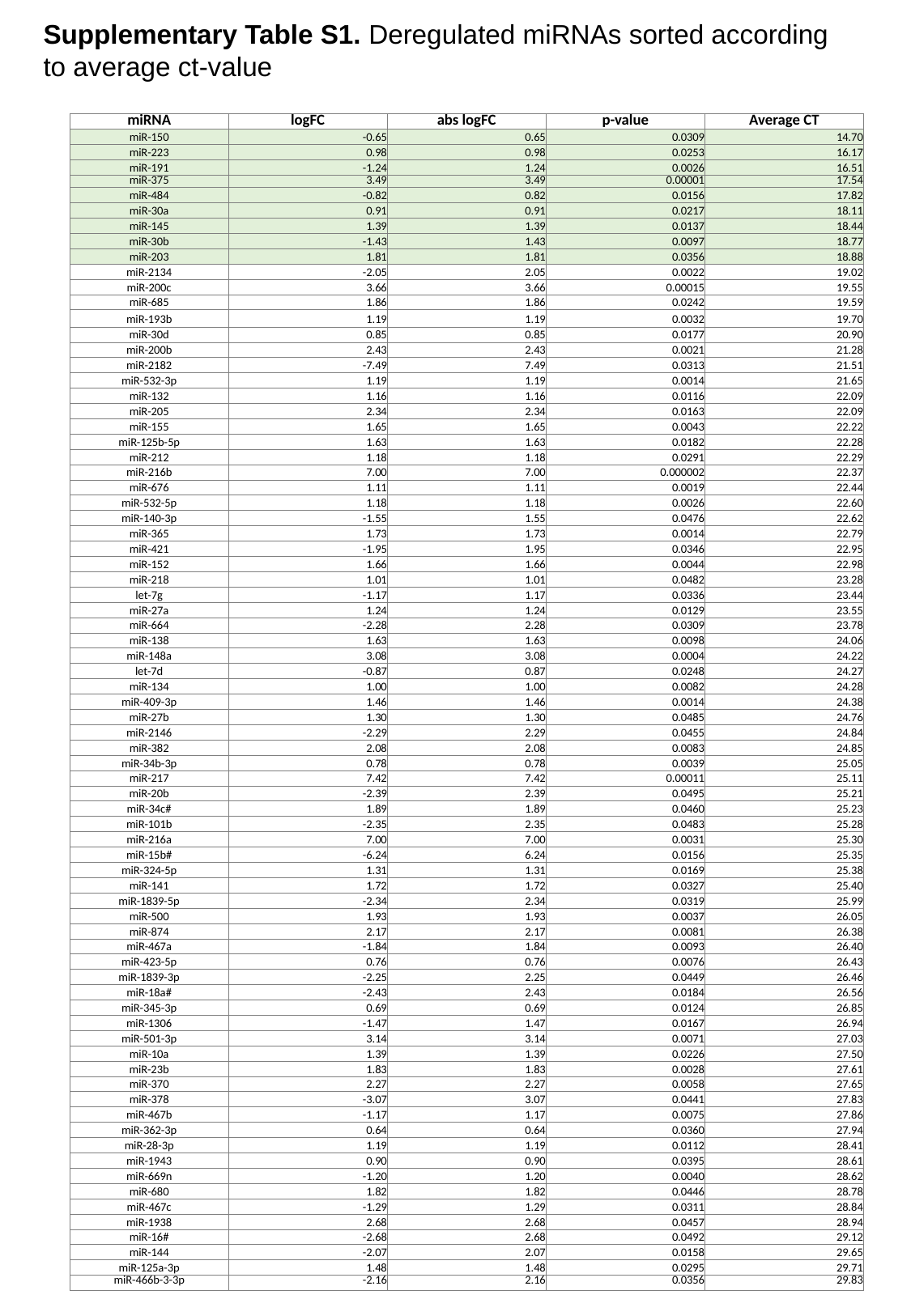

Supplementary Table S1. Deregulated miRNAs sorted according
to average ct-value
| miRNA | logFC | abs logFC | p-value | Average CT |
| --- | --- | --- | --- | --- |
| miR-150 | -0.65 | 0.65 | 0.0309 | 14.70 |
| miR-223 | 0.98 | 0.98 | 0.0253 | 16.17 |
| miR-191 | -1.24 | 1.24 | 0.0026 | 16.51 |
| miR-375 | 3.49 | 3.49 | 0.00001 | 17.54 |
| miR-484 | -0.82 | 0.82 | 0.0156 | 17.82 |
| miR-30a | 0.91 | 0.91 | 0.0217 | 18.11 |
| miR-145 | 1.39 | 1.39 | 0.0137 | 18.44 |
| miR-30b | -1.43 | 1.43 | 0.0097 | 18.77 |
| miR-203 | 1.81 | 1.81 | 0.0356 | 18.88 |
| miR-2134 | -2.05 | 2.05 | 0.0022 | 19.02 |
| miR-200c | 3.66 | 3.66 | 0.00015 | 19.55 |
| miR-685 | 1.86 | 1.86 | 0.0242 | 19.59 |
| miR-193b | 1.19 | 1.19 | 0.0032 | 19.70 |
| miR-30d | 0.85 | 0.85 | 0.0177 | 20.90 |
| miR-200b | 2.43 | 2.43 | 0.0021 | 21.28 |
| miR-2182 | -7.49 | 7.49 | 0.0313 | 21.51 |
| miR-532-3p | 1.19 | 1.19 | 0.0014 | 21.65 |
| miR-132 | 1.16 | 1.16 | 0.0116 | 22.09 |
| miR-205 | 2.34 | 2.34 | 0.0163 | 22.09 |
| miR-155 | 1.65 | 1.65 | 0.0043 | 22.22 |
| miR-125b-5p | 1.63 | 1.63 | 0.0182 | 22.28 |
| miR-212 | 1.18 | 1.18 | 0.0291 | 22.29 |
| miR-216b | 7.00 | 7.00 | 0.000002 | 22.37 |
| miR-676 | 1.11 | 1.11 | 0.0019 | 22.44 |
| miR-532-5p | 1.18 | 1.18 | 0.0026 | 22.60 |
| miR-140-3p | -1.55 | 1.55 | 0.0476 | 22.62 |
| miR-365 | 1.73 | 1.73 | 0.0014 | 22.79 |
| miR-421 | -1.95 | 1.95 | 0.0346 | 22.95 |
| miR-152 | 1.66 | 1.66 | 0.0044 | 22.98 |
| miR-218 | 1.01 | 1.01 | 0.0482 | 23.28 |
| let-7g | -1.17 | 1.17 | 0.0336 | 23.44 |
| miR-27a | 1.24 | 1.24 | 0.0129 | 23.55 |
| miR-664 | -2.28 | 2.28 | 0.0309 | 23.78 |
| miR-138 | 1.63 | 1.63 | 0.0098 | 24.06 |
| miR-148a | 3.08 | 3.08 | 0.0004 | 24.22 |
| let-7d | -0.87 | 0.87 | 0.0248 | 24.27 |
| miR-134 | 1.00 | 1.00 | 0.0082 | 24.28 |
| miR-409-3p | 1.46 | 1.46 | 0.0014 | 24.38 |
| miR-27b | 1.30 | 1.30 | 0.0485 | 24.76 |
| miR-2146 | -2.29 | 2.29 | 0.0455 | 24.84 |
| miR-382 | 2.08 | 2.08 | 0.0083 | 24.85 |
| miR-34b-3p | 0.78 | 0.78 | 0.0039 | 25.05 |
| miR-217 | 7.42 | 7.42 | 0.00011 | 25.11 |
| miR-20b | -2.39 | 2.39 | 0.0495 | 25.21 |
| miR-34c# | 1.89 | 1.89 | 0.0460 | 25.23 |
| miR-101b | -2.35 | 2.35 | 0.0483 | 25.28 |
| miR-216a | 7.00 | 7.00 | 0.0031 | 25.30 |
| miR-15b# | -6.24 | 6.24 | 0.0156 | 25.35 |
| miR-324-5p | 1.31 | 1.31 | 0.0169 | 25.38 |
| miR-141 | 1.72 | 1.72 | 0.0327 | 25.40 |
| miR-1839-5p | -2.34 | 2.34 | 0.0319 | 25.99 |
| miR-500 | 1.93 | 1.93 | 0.0037 | 26.05 |
| miR-874 | 2.17 | 2.17 | 0.0081 | 26.38 |
| miR-467a | -1.84 | 1.84 | 0.0093 | 26.40 |
| miR-423-5p | 0.76 | 0.76 | 0.0076 | 26.43 |
| miR-1839-3p | -2.25 | 2.25 | 0.0449 | 26.46 |
| miR-18a# | -2.43 | 2.43 | 0.0184 | 26.56 |
| miR-345-3p | 0.69 | 0.69 | 0.0124 | 26.85 |
| miR-1306 | -1.47 | 1.47 | 0.0167 | 26.94 |
| miR-501-3p | 3.14 | 3.14 | 0.0071 | 27.03 |
| miR-10a | 1.39 | 1.39 | 0.0226 | 27.50 |
| miR-23b | 1.83 | 1.83 | 0.0028 | 27.61 |
| miR-370 | 2.27 | 2.27 | 0.0058 | 27.65 |
| miR-378 | -3.07 | 3.07 | 0.0441 | 27.83 |
| miR-467b | -1.17 | 1.17 | 0.0075 | 27.86 |
| miR-362-3p | 0.64 | 0.64 | 0.0360 | 27.94 |
| miR-28-3p | 1.19 | 1.19 | 0.0112 | 28.41 |
| miR-1943 | 0.90 | 0.90 | 0.0395 | 28.61 |
| miR-669n | -1.20 | 1.20 | 0.0040 | 28.62 |
| miR-680 | 1.82 | 1.82 | 0.0446 | 28.78 |
| miR-467c | -1.29 | 1.29 | 0.0311 | 28.84 |
| miR-1938 | 2.68 | 2.68 | 0.0457 | 28.94 |
| miR-16# | -2.68 | 2.68 | 0.0492 | 29.12 |
| miR-144 | -2.07 | 2.07 | 0.0158 | 29.65 |
| miR-125a-3p | 1.48 | 1.48 | 0.0295 | 29.71 |
| miR-466b-3-3p | -2.16 | 2.16 | 0.0356 | 29.83 |

## Slide 4
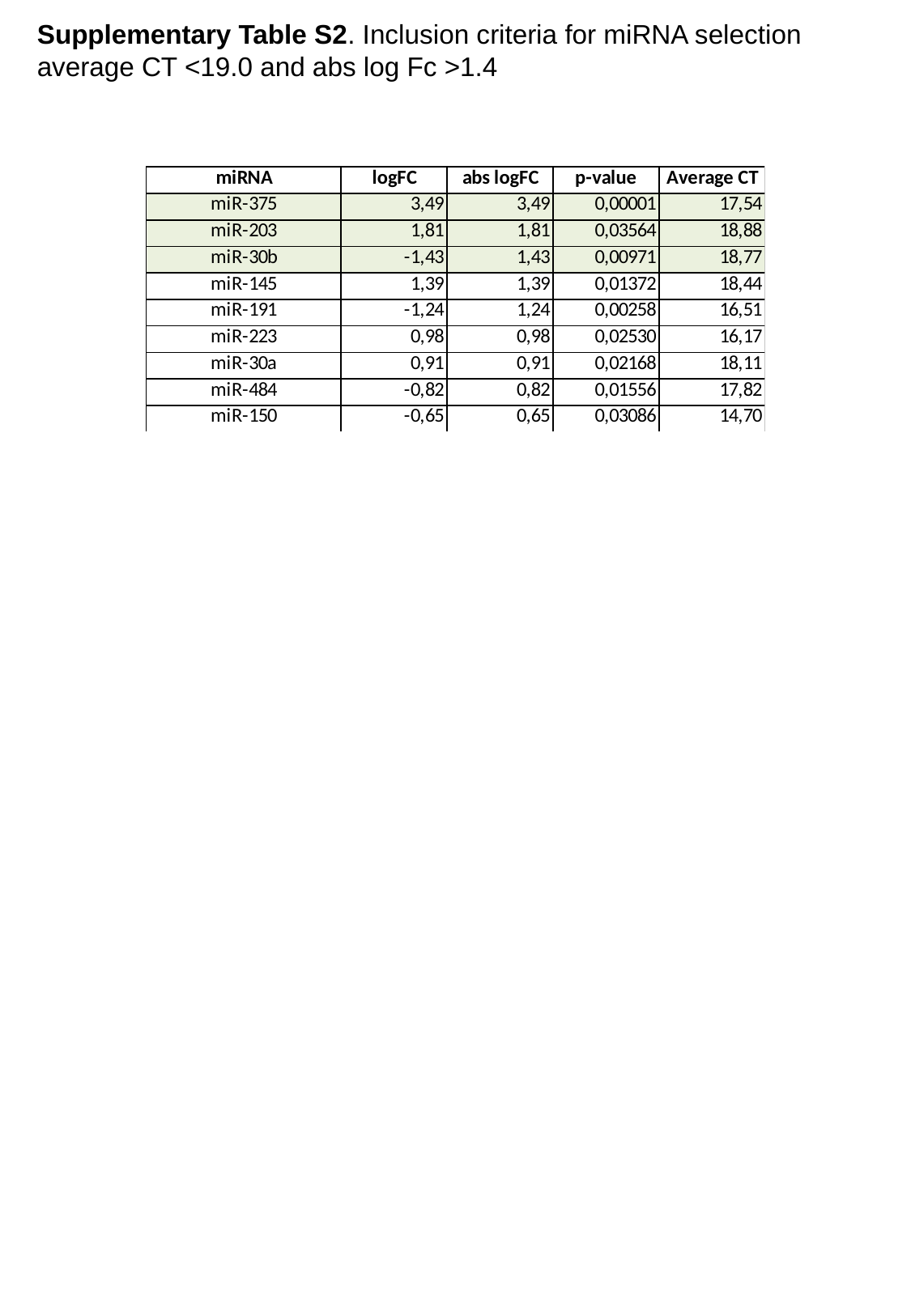

Supplementary Table S2. Inclusion criteria for miRNA selection average CT <19.0 and abs log Fc >1.4

## Slide 5
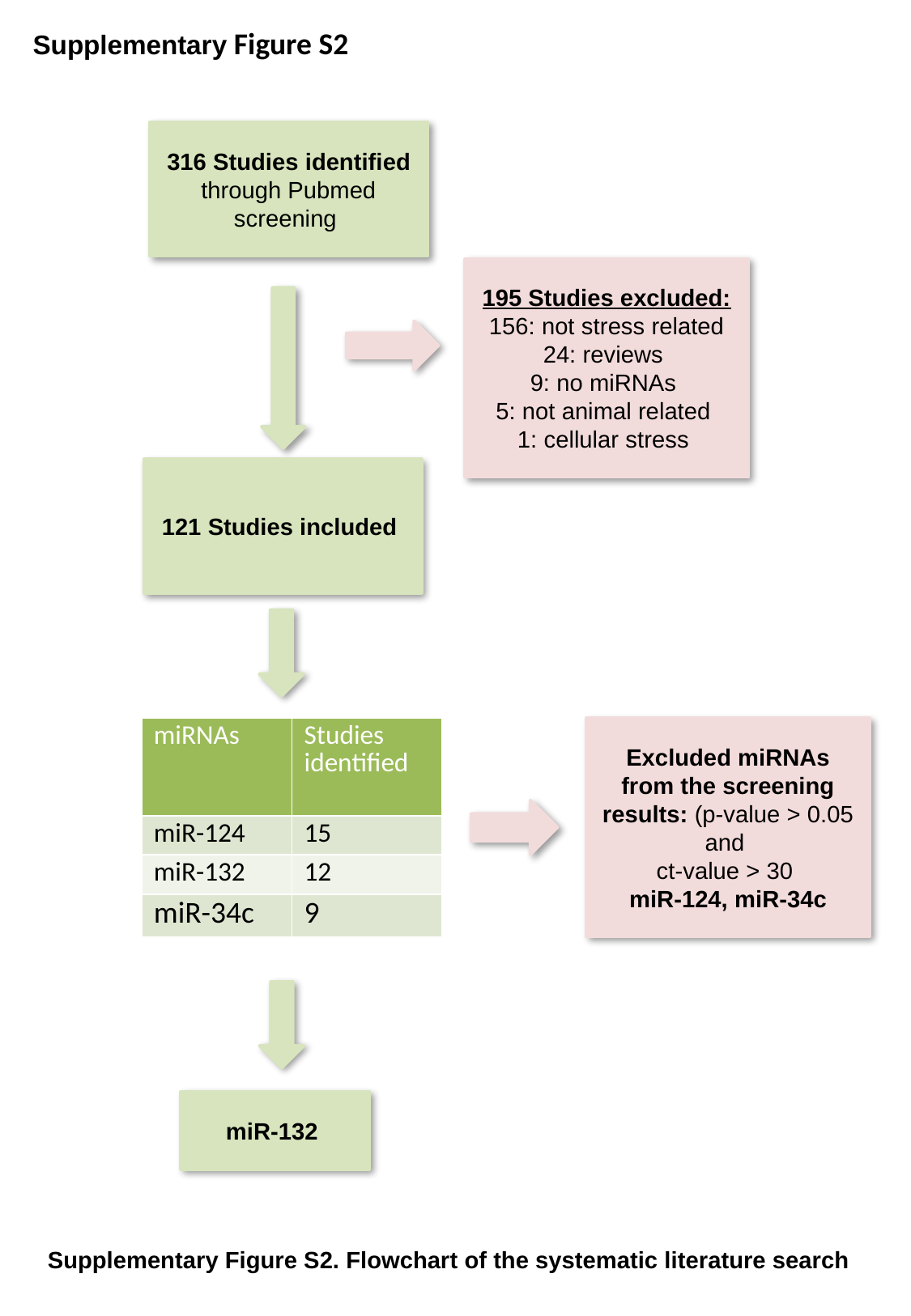

Supplementary Figure S2
316 Studies identified through Pubmed screening
195 Studies excluded:
156: not stress related
24: reviews
9: no miRNAs
5: not animal related
1: cellular stress
121 Studies included
Excluded miRNAs from the screening results: (p-value > 0.05 and
ct-value > 30
miR-124, miR-34c
| miRNAs | Studies identified |
| --- | --- |
| miR-124 | 15 |
| miR-132 | 12 |
| miR-34c | 9 |
miR-132
Supplementary Figure S2. Flowchart of the systematic literature search
